# Effects of cruciate embedding fascia-bone flap technique on grade II–III cerebral spinal fluid leak in endoscopic endonasal surgery

**DOI:** 10.1186/s12893-022-01730-9

**Published:** 2022-07-26

**Authors:** WenJi Zhao, Gang Yang, RuiChun Li, Gang Huo, Dong Gao, MingChuan Cao, XiaoShu Wang

**Affiliations:** 1 Department of Neurosurgery, The First Affiliated Hospital of Chongqing Medical University, 1# Youyi Road, Yuzhong District, Chongqing, 400016 People’s Republic of China; 2 Department of Neurosurgery, The First Affiliated Hospital of Xi’an Jiaotong University, 277 West Yanta Road, Xi’an, 710061 People’s Republic of China

**Keywords:** Endoscopic endonasal surgery, CSF leak, Bone flap, Skull base reconstruction, Pedicle vascularized nasoseptal flap

## Abstract

**Background:**

Cerebral spinal fluid (CSF) leak remains an important issue in endoscopic endonasal surgery (EES). A standard protocol for skull base closure has not yet been established, and the application of rigid buttress has not been given sufficient attention. To emphasize the functions of support and fixation from rigid buttress in reconstruction, we introduced the cruciate embedding fascia-bone flap (CEFB) technique using autologous bone graft to buttress the fascia lata attachment to the partially sutured skull base dural defect and evaluated its efficacy in a consecutive case series of grade II–III CSF leaks in EES.

**Methods:**

Data from consecutive patients diagnosed with sellar region lesions with grade II–III CSF leaks during EES were collected from May 2015 to May 2020. Skull base reconstructions were performed with the CEFB or the conventional pedicle vascularized nasoseptal flap (PNSF). Related clinical data were analysed. The combined use of the CEFB and PNSF was applied to an additional supplemental case series of patients with grade III leak and multiple high-risk factors.

**Results:**

There were 110 and 65 patients included in the CEFB and PNSF groups, respectively. The CEFB demonstrated similar effects on the incidence of postoperative CSF leak (2.7%), intracranial infection (4.5%), and lumbar drainage (LD) placement (5.5%) as PNSF (3.1%, 3.1%, and 6.2%), but with less epistaxis (CEFB: 0%, PNSF: 6.2%) and nasal discomforts (CEFB: 0%, PNSF: 7.7%). The LD duration (CEFB: 6.67 ± 2.16 days, PNSF: 10.50 ± 2.38 days), bed-stay time (CEFB: 5.74 ± 1.58 days, PNSF: 8.83 ± 3.78 days) and hospitalization time (CEFB: 10.49 ± 5.51 days, PNSF: 13.58 ± 5.50 days) were shortened in the CEFB group. The combined use of CEFB and PNSF resulted in 0 postoperative CSF leaks in the supplemental case series of 23 highly susceptible patients.

**Conclusion:**

This study suggested that the new CEFB technique has the potential to prevent postoperative CSF leak in EES. The results indicated that it can be used effectively without PNSF in suitable cases or applied in addition to a PNSF with high compatibility when necessary. Its effectiveness should be further verified with a larger cohort and better design in the next step.

*Trial Registration* Current Controlled Trials ChiCTR2100044764 (Chinese Clinical Trial Registry); date of registration: 27 March 2020. Retrospectively registered

## Background

Endoscopic endonasal surgery (EES) has become a popular method for resecting ventral skull base tumours with minimal invasiveness and good visibility [1–3]. However, the risk of postoperative cerebral spinal fluid (CSF) leak remains an issue due to the challenge in achieving watertight closure of the skull base [4]. The incidence of CSF leak after EES has been reported to vary from 1.6 to 40% [5–12]. Meningitis, pneumocephalus, and other complications have a great impact on the prognosis of patients [5, 13]. Although various skull base reconstruction methods have emerged, a standard protocol has not yet been established. The introduction of the pedicle vascularized nasoseptal flap (PNSF) has dramatically improved the outcome of skull base repairs since 2006 [14]. It has become a well-accepted and even predominant procedure for high-flow CSF leaks in EES. But the PNSF involves distinct anatomical transposition of the nasal mucosa and may cause complications including perforation, epistaxis, dysosmia, and nasal discomforts affecting quality of life [15–17]. It emphasizes soft (membranous) reconstruction but lacks rigid support of the skull base. Methods represented by gasket-seal [6] and in situ bone flap (ISBF) [18] have proposed rigid buttress in addition to soft reconstruction and have achieved satisfactory outcomes in preventing postoperative CSF leaks. However, artificial grafts, PNSF, lumbar drainage (LD), and iodoform gauze nasal packing are still indispensable in these applications [6, 16, 17, 19].

We have been applying the cruciate embedding fascia-bone flap (CEFB) technique in suitable cases of intraoperative grade II–III CSF leak since 2015, with the purpose of integrating soft-tissue repair, rigid buttress, and multilayer reconstruction. We have aimed to restore the normal anatomical layers of the skull base by using autologous material and with less nasal interference. Another feature we have targeted is the independent use of the CEFB flap without routine PNSF, iodoform gauze and LD, although it can be completely PNSF-compatible when necessary. In this article, we described this new skull base repair method in detail and assessed its efficacy in a case series of consecutive patients.

## Materials and methods

### Patients and group

**Fig. 1:**
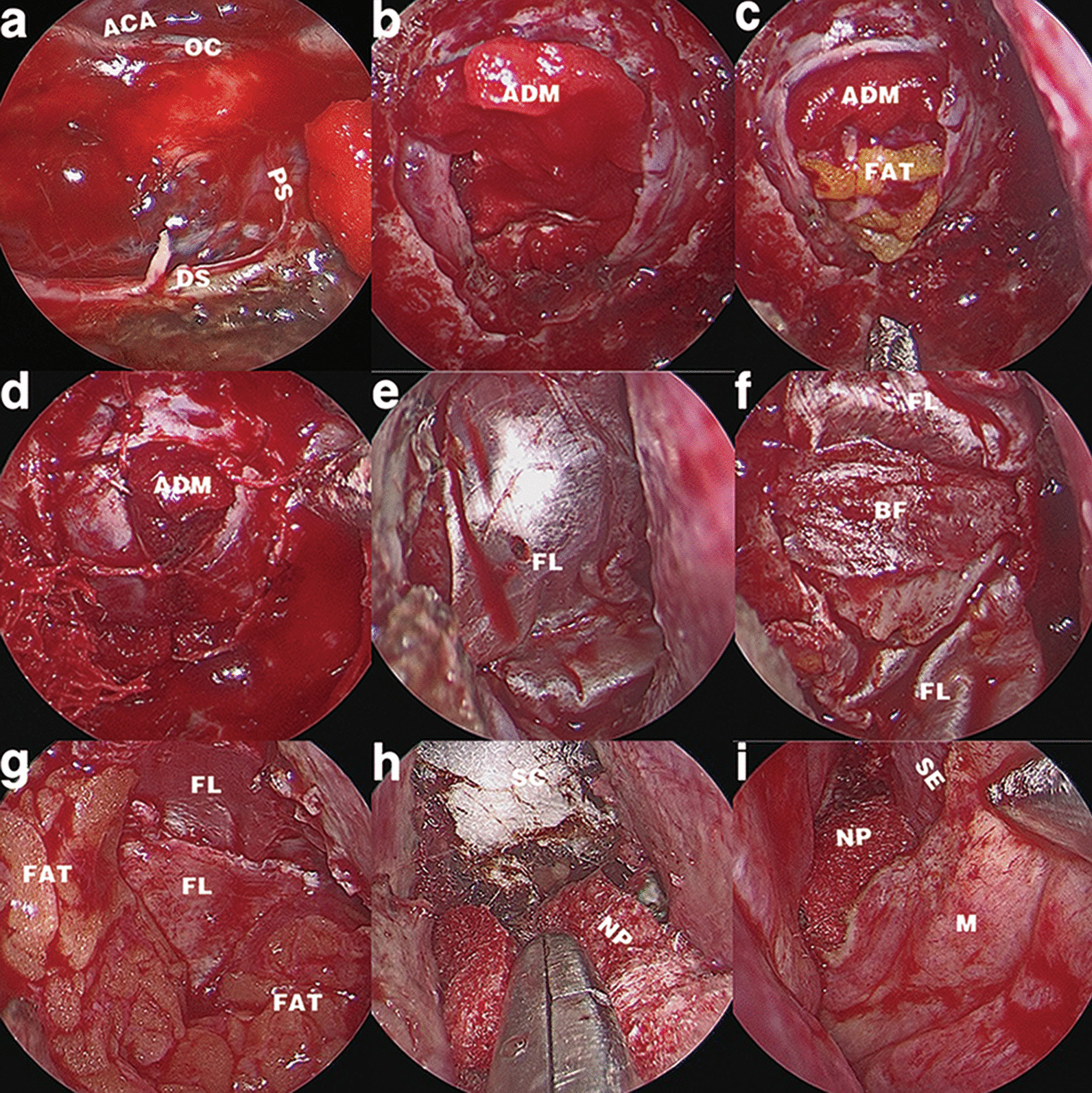
Representative intraoperative images of the CEFB procedures. **a** After removal of a giant pituitary adenoma breaching the diaphragma sellae, a grade III CSF leak is observed. **b** Absorbable ADM is placed to cover the margin of the residual diaphragma sellae as the first subdural inlay. **c** An optimal amount of autologous fat graft is placed inside the sellar space to sustain the ADM and generate appropriate tension to fit the following steps for the rigid buttress. **d** Partial dural suturing with 3 stitches was applied on the “Y”-shaped dural incision to reduce the dural defect and confine it under the centre of the rigid buttress. **e** An onlay of fascia lata is longitudinally placed to cover the dural defect with a redundancy of 10 mm on the front and rear ends. The lateral edges of the fascia slightly exceed the lateral bone defect margin. **f** A bone flap graft is transversely embedded under the lateral defect edges to buttress the longitudinally placed fascia underneath, forming a cruciate embedding complex. The fascia can stretch out through the frontal and rear gaps between the bone flap and defect edge. **g** Surplus grafts of fascia and fat are used to cover and strengthen the entirety of the CEFB constructs. **h** Surgicel and Nasopore are placed inside the sphenoid sinus to fix and support the fat and fascia. **i** The nasal mucosa is repositioned back to the septum without formation of the PNSF. *ACA* anterior cerebral artery, *ADM* acellular dermis matrix, *OC* optic chiasm, *BF* bone flap, *FL* fascia lata, *DS* diaphragma sellae, *PS* pituitary stalk, *SC* surgicel, *NP* nasopore, *M* mucosa, *SE* septum

Under approval of the institutional ethics committee, we retrospectively collected data from consecutive patients diagnosed with sellar region lesions who underwent EES by the same group of senior surgeons in our institution from May 2015 to May 2020. All operations were in accordance with the general surgical indications for sellar regional lesions: neurological impairment due to lesional compression, progressive lesion growth, functioning adenoma resistant to drug therapy, and increased intracranial pressure induced by lesional mass effects. All patients were confirmed to have an intraoperative grade II or III CSF leak corresponding to Esposito’s Grade [20] (Fig. 1a), and the formation mechanism of their leaks was identical: skull base bone-dura defects.

In the reconstruction phase of the operation, patients who received CEFB were included as the CEFB group; patients with insufficient bone harvest for CEFB (this anatomical variation, encountered in the nasal phase of the surgical approach, has not been considered an influencing factor of postoperative CSF leak after EES [21, 22]) were treated with PNSF and served as the PNSF group. Data regarding age, sex, body mass index (BMI), diabetes, lesion volume, smoking, hypertension, surgical approach and pathology were analysed to verify the consistency of the baseline between the two groups.

Patients who simultaneously had grade III intraoperative CSF leak and $\ge$ 3 high-risk factors for postoperative CSF leak (diabetes, age > 65, hydrocephalus, body mass index (BMI) > 30, adoption of extended approach [23]) received a combination of CEFB and PNSF to strengthen the reconstruction. As a supplemental case series, we also recorded and described the outcomes of these patients.

We excluded recurrent tumour patients due to their compromised tissue healing ability, as local cicatrization would impair the reconstruction [24], causing confounding fluctuations in the data. All patients enrolled were followed-up for at least 6 months, otherwise, they were excluded.

### Surgical technique

The two-surgeon, four-hand technique was used in all operations with a high-definition endoscope (Karl Storz, Tuttlingen, Germany). The standard endoscopic endonasal approach (EEA) was mainly adopted in cases of pituitary adenoma and Rathke cleft cyst, and the extended EEA (EEEA) was mainly used in operations on craniopharyngioma, meningioma, and invasive or giant pituitary adenoma. The multilayer closure strategy was applied in all operations.

### Inlay procedures

The inlay parts in both groups were identical. Absorbable acellular dermal matrix (ADM) (Heal-all, ZH-BIO Inc., Yantai, China) was used as the first subdural inlay. It was placed to cover the margin of the residual diaphragma sellae (Fig. 1b). In cases of total loss of diaphragma sellae, the ADM was placed at the original site of the diaphragma sellae and meticulously leaned on structures such as the optic chiasm and hypophyseal stalk or tucked under the internal carotid artery (ICA). Then, an autologous fat graft harvested from the patient’s thigh was placed inside the sellar space or corresponding subdural space under the ADM to sustain the first layer (Fig. 1c).

### Partial dural suturing

Interrupted partial dural suturing was applied in all operations. The suturing was made with 5–0 nylon by the sliding-lock-knot technique as described previously [8]. The dural incision was preferably made in an “H”, “T”, “Y”, or inverted “U” shape to facilitate the multipoint suturing of 3–5 stitches (Fig. 1d).

### Onlay of the fascia lata

In the CEFB group, the fascia lata onlay was implanted according to the following protocols. First, the fascia lata was tailored into a rectangle shape and placed over the dural defect either longitudinally (sagittal direction) or transversely (coronary direction). We preferred the longitudinal onlay in most cases for better convenience. Typically, in extended EEA, the frontal edge of the fascia lata reaches the planum sphenoidale, and the rear edge reaches the upper clivus, extending at least 10 mm beyond the bone defect edges. The lateral edges of the fascia should reach the lateral bone defect margin or slightly exceed it by no more than 2 mm (Fig. 1e).

In the PNSF group, the onlay of the fascia lata was performed in a conventional way by covering the bone defect with a redundancy of at least 10 mm.

### Embedding of bone flap graft

In the CEFB group, the bone flap graft was harvested from the nasal septum, vomer, anterior wall of the sphenoid sinus, or bone septum inside the sphenoid sinus. The bone flap was countersunk and embedded under the edges of the bone defect as a rigid buttress to press the fascia lata onto the dural defect. It should be noted that the bone flap embedding was different from the circumferential wedging used in gasket-seal. In the CEFB procedure, the flap was wedged on 2 sides either longitudinally or transversely and placed vertically, crossing upon the fascia lata to finally form a cruciate embedding fascia-bone flap complex. The bone flap was trimmed and wedged with caution to avoid neurovascular injury. Typically, we transversely embed (vertical to the longitudinally placed fascia underneath) the bone flap under the lateral defect edges with an overlap exceeding 1–2 mm (avoiding compression on the optic nerve or ICA). Thus, the fascia could stretch out through the frontal and rear gaps between the bone flap and defect edge and be paved smoothly onto the skull base (Fig. 1f).

In the CEFB group, surplus fascia and fat graft were used to strengthen the whole construct (Fig. 1g). The out-stretching fascia was fixed with oxidized regenerated cellulose (Surgicel, Ethicon Inc., America) for apposition against the bone surface. The sphenoid sinus was filled with absorbable lactide caprolactone copolyesters (Nasopore, Polyganics, Groningen, The Netherlands) to sustain the fascia (Fig. 1h), and similarly, the nasal cavity was filled to reposition the mucosa back to the septum. No glue, balloon or iodoform gauze was used in the CEFB group (Fig. 1i).

### PNSF application

In the PNSF group, a vascularized nasoseptal flap was harvested as described previously [14] to cover the fascia abundantly as the last layer. Surplus fat graft and iodoform gauze were inserted into the sphenoid sinus and nasal cavity to support PNSF coverage.

### Postoperative management

In both groups, the supine position was mandatory in the first postoperative 24 h. Then, the head of the bed was gradually elevated 20–30° per day unless a CSF leak was detected. The patient was allowed to get out of bed after no CSF leakage while in a persistent vertical sitting posture for 4 h. Lumbar drainage (LD) was not prophylactically used but was only performed when the patient had sustained CSF leak or a refractory intracranial infection. The nasal packing gauze in the PNSF group was removed 10 days after surgery.

### Collection of intraoperative and postoperative data

Data regarding surgery duration, resection degree, grade/size of the CSF leak, intracranial infection, LD placement, LD duration, epistaxis, dysosmia, nasal discomforts, bed-stay time and hospitalization time were recorded for comparison. Nasal discomforts was introduced as a subjective symptomological item that was evacuated 10 weeks after surgery, including senses of dryness, pain, nasal obstruction, runny nose and nasal odour.

### Statistical analysis

For continuous variables (age, BMI, lesion volume, leak size, surgical duration, etc.), Student’s t test was used for comparisons between the CEFB and PNSF groups. For categorical variables (sex, diabetes, smoking, hypertension, surgical approach, pathology, leak grade, CSF leak and infection, etc.), the Chi-squared test or Fisher’s exact test was used for comparison.

SPSS 25.0 was used for analyses. For the baseline data, a p value greater than 0.1 suggested that the difference in a variable between the two groups was uncertain. For the related indicators describing the effects of reconstruction, a p value less than 0.05 indicated the existence of a difference between groups.

## Results

### Baseline characteristics

Three patients were excluded from the study due to loss to follow-up (1 in CEFB and 2 in PNSF; total loss rate of follow-up 1.7%). None of them presented with CSF leak or meningitis in their last interview after surgery, but we then lost contact with them. The included patients were followed-up for at least 6 months. Finally, there were 110 patients enrolled in the CEFB group and 65 in the PNSF group.

For the preoperative baseline parameters, there were no significant differences between the 2 groups in age, sex, BMI, diabetes, smoking, hypertension, surgical approach or pathology of the lesions (p > 0.1) (Table 1). Despite the p > 0.1 result, due to the existence of type II errors, it cannot be simply asserted that the baselines of the two groups were completely consistent. However, the baselines of the two groups could be considered similar to a certain degree.

**Table 1:** Comparison of baseline characteristics

Characteristics	CEFB	PNSF
No. of patients	110	65
Age at surgery	50.12 (± 10.96)	49.60 (± 9.14)
(years, mean ± SD)		
Gender		
Male (%)	62 (56.4%)	36 (55.4%)
Female (%)	48 (43.6%)	29 (44.6%)
BMI (kg/m^2^, mean ± SD)	23.09 (± 3.35)	23.27(± 2.66)
Diabetes (%)	10 (9.1%)	7 (10.8%)
Smoking (%)	52 (47.3%)	31 (47.7%)
Hypertension (%)	36 (32.7%)	24 (36.9%)
Surgical approach		
EEA (%)	72 (64.5%)	39 (60.0%)
EEEA (%)	38 (34.5%)	26 (40.0%)
Lesion volume (cm^3^, mean ± SD)	7.83 (± 2.04)	7.75(± 2.47)
Pathology		
Pituitary adenoma (%)	70 (63.6%)	41 (63.1%)
Craniopharyngioma (%)	20 (18.2%)	15 (23.1%)
Rathke Cyst (%)	8 (7.3%)	3 (4.6%)
Arachnoid Cyst (%)	4 (3.6%)	1 (1.5%)
Meningioma (%)	8 (7.3%)	5 (7.7%)

*BMI* body mass index, *EEA* endoscopic endonasal approach, *EEEA* extended endoscopic endonasal approach

### Intraoperative and postoperative conditions

The grade II/III constituent ratio and size of intraoperative CSF leak (69/41 vs. 33/32; 18.55 ± 2.41 vs. 18.38 ± 3.09 mm^2^, p > 0.1), incidence of postoperative CSF leak (2.7% vs. 3.1%, p > 0.1), intracranial infection (4.5% vs. 3.1%, p > 0.1) and LD placement (5.5% vs. 6.2%, p > 0.1) were similar between the CEFB and PNSF groups.

The CEFB group had a shorter LD duration (6.67 ± 2.16 vs. 10.50 ± 2.38 days, p < 0.01), bed-stay time (5.74 ± 1.58 vs. 8.83 ± 3.78 days, p < 0.01) and hospitalization time (10.49 ± 5.51 vs. 13.58 ± 5.50 days, p < 0.01) than the PNSF group. The CEFB was also associated with a lower occurrence of epistaxis (0 vs. 6.2%, p < 0.05) and nasal discomforts (0 vs. 7.7%, p < 0.01). However, the surgery duration of the CEFB group was longer than that of the PNSF group (2.62 ± 0.56 vs. 2.26 ± 0.62 h, p < 0.05), which was mainly ascribed to the meticulous trimming and embedding of the bone flap graft. All comparisons above are listed in Table 2.

**Table 2:** Intraoperative and postoperative characteristics

Characteristics	CEFB	PNSF
No. of patients	110	65
Leak grade		
Grade II (%)	69 (62.7%)	33 (50.8%)
Grade III (%)	41 (37.3%)	32 (49.2%)
Leak size	18.55 (± 2.41)	18.38 (± 3.09)
(mm^2^,mean ± SD)		
Gross total resection (%)	100 (90.9%)	58 (89.2)
Surgery duration	2.62 (± 0.56)	2.26 (± 0.62)
(hours, mean ± SD)		
Postoperative CSF leak (%)	3 (2.7%)	2 (3.1%)
Infection (%)	5 (4.5%)	2 (3.1%)
LD placement (%)	6 (6.5%)	4 (6.2%)
LD duration (days, mean ± SD)	6.67 (± 2.16)	10.50(± 2.38)
Epistaxis (%)	0 (0)	4 (6.2%)
Dysosmia (%)	1 (0.9%)	3 (4.6%)
Nasal discomforts (%)	0 (0)	5 (7.7%)
Bed stay time	5.74 (± 1.58)	8.83(± 3.79)
(days, mean SD)		
Hospitalization time	10.49 (± 5.51)	13.58(± 5.50)
(days, mean ± SD)		

*LD* lumbar drainage

During the process of bone flap embedding, a small amount of epidural haemorrhages often occurred but could be easily controlled by a gelatine sponge. No CEFB-related neurovascular injury occurred. One patient in the CEFB group and another in the PNSF group who experienced sustained postoperative CSF leak were cured by reoperation. The one in the CEFB group was caused by local curling of the fascia lata due to excessive compression of the bone flap. Another in the PNSF group was due to loosening of the PNSF. Other cases of postoperative CSF leak were healed by LD placement and postural confinement.

**Fig. 2:**
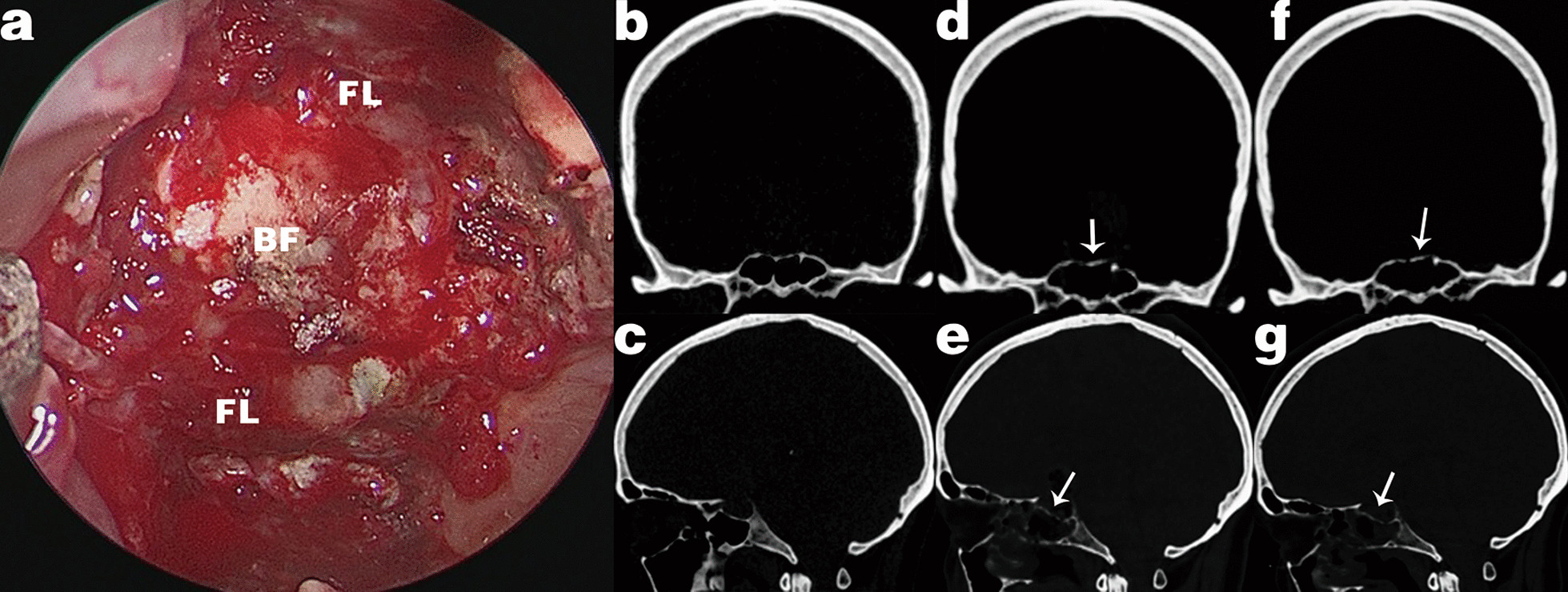
Representative postoperative images of CEFB outcomes. **a** During debridement under endoscopy 3 weeks after surgery, the bone flap and fascia are found to be in place and firmly attached to the defect. **b** Preoperative coronal and **c** sagittal CT images of the skull base bone structure. **d** Immediate postoperative coronal and **e** sagittal CT images of CEFB reconstruction. **f** Three months after surgery, coronal and **g** sagittal CT images demonstrate no dislocation or detachment of the bone flap. *BF* bone flap, *FL* fascia lata, Arrowhead = bone flap graft

No fracture, dislocation or detachment of the CEFB construct was observed during the scheduled nasal debridement under endoscopy 3 weeks after surgery (Fig. 2a). The bone flap and fascia were found to be in place and firmly attached to the defect. Osteal structure reconstruction was assessed by pre- and postoperative CT scans (Fig. 2b–e). The long-term stability of the CEFB construct was examined by CT scan 3–6 months after surgery (Fig. 2f, g).

### Subdivided comparisons

Data on subdivided characteristics are summarized in Table 3. In both grade II and grade III leak comparisons, the CEFB group showed similar effects as the PNSF group on the incidence of postoperative CSF leak (grade II leak: 2.9% vs. 3.0%; grade III leak: 2.4% vs. 3.1%), infection (grade II leak: 4.3% vs. 3.0%; grade III leak: 4.9% vs. 3.1%), and LD placement (grade II leak: 4.3% vs. 6.1%; grade III leak: 7.3% vs. 6.3%).

**Table 3:** Subdivided characteristics comparisons

Subdivided characteristics		CEFB	PNSF
Grade II leakage	No. of patients	69	33
	Postoperative CSF leak (%)	2 (2.9%)	1 (3.0%)
	Infection (%)	3 (4.3%)	1 (3.0%)
	LD placement (%)	3 (4.3%)	2 (6.1%)
Grade III leakage	No. of patients	41	32
	Postoperative CSF leak (%)	1 (2.4%)	1 (3.1%)
	Infection (%)	2 (4.9%)	1 (3.1%)
	LD placement (%)	3 (7.3%)	2 (6.3%)
Preoperative hydrocephalus	No. of patients	5	3
	Postoperative CSF leak (%)	0 (0)	1 (33.3%)
	Infection	1 (20.0%)	0 (0)
	LD placement (%)	1 (20.0%)	1 (33.3%)

*LD* lumbar drainage

Among patients with preoperative hydrocephalus, the CEFB group yielded an outcome of 0 postoperative CSF leak out of 5 patients, while 1 leak occurred among 3 patients in the PNSF group.

### Additional supplementary cases

There were 23 patients who had grade III intraoperative CSF leaks and multiple high-risk factors (as described in the Patients and group section) received the combined use of CEFB and PNSF. Ten patients had preoperative hydrocephalus. There was no postoperative CSF leak or LD placement, but 2 patients experienced postoperative infection.

## Discussion

### Benefits of inlay and partial dural suturing in the CEFB technique

The inlay materials contribute to reducing the leak size and CSF flow, eliminating dead space, buffering the impact of CSF pulsation, and alleviating CSF pooling or soaking [25, 26]. The key point of the CEFB inlay procedure is to finely adjust the volume of the fat graft to obtain optimal subdural tension fitting the buttress pressure of the wedged bone graft, generating appropriate tightness of the attachment between the fascia and dura. Deep suturing and knotting are no longer major issues in EES today, but literally “watertight” suturing is still technically challenging due to dural dehydration, fragility, or electrocauterization [8, 27]. Partial suturing is not sufficient to seal CSF leaks, but we deem it to still have the following benefits: 1. The dural defect is reduced and confined under the centre of the rigid buttress. 2. The dural interface is made available for onlay fascia attachment, avoiding direct contact between the fascia and inlay grafts. 3. The ADM and fat are anchored in place. 4. Intrasellar tension and compactness are increased [28].

### Comparison between the CEFB procedure and gasket-seal

**Fig. 3:**
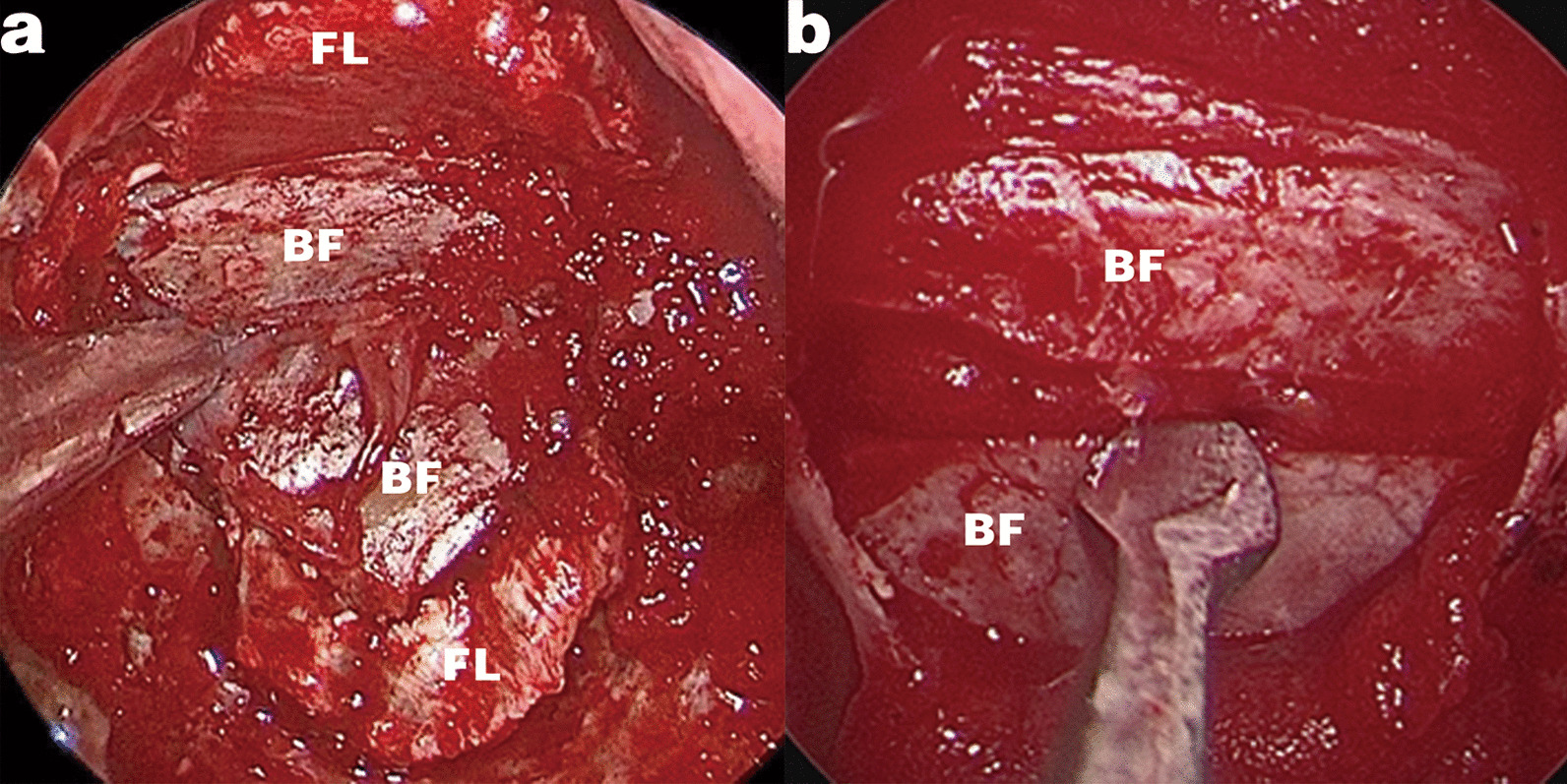
Representative intraoperative images of CEFB variants. **a** Two separated bone flap grafts are embedded at the planum sphenoidale and sellar floor respectively, buttressing the fascia in different directions on angled planes. **b** The bone graft is tailored into narrow strips and then wedged at intervals onto the defect for economical use of the limited bone graft harvest. *BF* bone flap, *FL* fascia lata

The CEFB procedure shares the same concept of rigid reconstruction with the gasket seal [6], but with significant modifications. As the name addressed, gasket-seal focuses on the watertight closure of defect. The shape of the circumferentially wedged material used in gasket-seal must be highly matched to the shape of the bone defect [6, 29]. Artificial material is frequently used [6, 19, 30] because the autologous bone graft does not always perfectly fit. The core of the CEFB technique is the buttress pressure firmly holding the fascia lata onto the dura to promote tight attachment and mutual adhesion. First, the bone flap in the CEFB construct is embedded at only two sides of the bone defect instead of being fully circumferentially wedged. The size of the bone flap is qualified by adequate length on only one axis, which promotes the usage of autologous bone, and brings lower costs and risks of infection or rejection. In our study, 67.2% (133/198) of all patients achieved satisfactory autologous bone harvest. Second, the two-side embedding of the bone flap allows the fascia lata to stretch out through the gaps on the non-wedged sides and to be paved smoothly onto skull base. However, when gasket-seal are used, the fascia lata forms a “cauliflower leaf” shape [6, 29] due to the tight depression in the centre [6]. The tilted or curled edge of the fascia makes it difficult to provide smooth attachment. Third, partial dural suturing ensures correct epidural embedding of the bone flap, avoiding accidental misplacement into the subdural space. The firm contact between the sutured dura and fascia also assists adhesion formation. Finally, the gasket-seal is not ideal in cases of defects traversing two geometric planes [6]. In our EEEA cases with sufficient bone graft harvest, we wedged two separated bone flaps at different defect planes respectively (Fig. 3a). The fascia lata could then be supported evenly and held in place on angled planes.

### Resistance of the CEFB construct against counteracting forces

Forces including brain gravity, CSF pulsation, and intracranial pressure are great concerns in skull base repair. These downwards forces are prone to compromising the reconstruction [28]. Hence, iodoform gauze packing, lumbar drainage, intranasal balloon, etc., are used as countermeasures [6, 30, 31]. The integrity of the CEFB construct is sturdy enough to resist these forces due to the rigidity of the firmly wedged bone flap. No dislocation or fracture of the bone flap was observed in any of our cases, even without iodoform gauze packing or lumbar drainage. These downwards forces might even be conjecturally helpful in inducing more solid compression of the CEFB structure and then strengthening the watertightness and attachment. This mechanism possibly contributed to the avoidance of CSF leaks among preoperative hydrocephalus patients and shortened the bed-stay time in the CEFB group. Especially in patients with preoperative hydrocephalus, both CEFB alone and in combination with PNSF exhibited a good outcome, with 0 postoperative CSF leaks in 15 patients.

### Considerations regarding the application of PNSF

The PNSF was a milestone in the development of skull base reconstruction due to its fast healing and long-term security of closure [14]. However, the harvesting of the PNSF necessitates a long incision on the nasal septum to mobilize an extensive piece of mucosa, which is then migrated to cover the skull base. The exposed donor site needs 6–12 weeks to be re-epithelialized [32]. Nasal complications related to this extensive shift of mucosa are not rare [15–17, 33]. The nasal packing of iodoform gauze or a balloon regularly used in association with PNSF also influences mucosa regeneration and the patients’ subjective experience due to intranasal pressure and stimulation [15]. Garcia-Navarro reported that the PNSF did not make a significant difference in their gasket-seal practice, which raised the question of PNSF necessity [6]. In our study, we obtained similar outcomes in terms of postoperative CSF leak and infection between the CEFB and PNSF groups, and nasal complications were even reduced in the CEFB group. The effectiveness of CEFB is also similar to that of other representative reports on postoperative CSF leak [6, 9, 18, 19] (Table 4). Our data suggest that with appropriate CEFB use and multilayer reconstruction, the PNSF may not be the sole mandatory option. The advantages and disadvantages of the PNSF should be individually weighed. We did not attempt to replace the PNSF with the CEFB. On the contrary, the CEFB technique is not exclusive but highly compatible with the PNSF. In 23 patients with grade III CSF leak and multiple high-risk factors, the combination of the 2 techniques achieved 0 postoperative CSF leaks. This implied that with the additional protection from the PNSF, the CEFB flap might provide reliable security in high-risk cases.

**Table 4:** Representative Literatures review of skull base repair

Authors and Year	Repair technique	Postoperative CSF leak rate		
		Overall	Grade 2	Grade 3
Garcia-Navarro et al. (2013) [6]	Gasket seal closure ± LD, ± PNSF	4.3% (2/46)	Data NA	4.3% (2/46)
Takayuki Ishikawa et al. (2018) [9]	Continuous dural suturing + fat graft + lactate plate ± PNSF	3.9% (3/76)	2.9%(1/34)	4.7% (2/42)
Andrew Conger et al. (2018) [19]	Fat + Collagen sponge + bone/ synthetic buttress + PNSF /sphenoid sinus mucosa	3.9% (7/181)	3.1%(3/98)	4.8% (4/83)
Biao Jin et al. (2020) [18]	ADM + ISBF ± fascia lata + PNSFs	2.1% (1/47)	Data NA	2.1% (1/47)
Present study	ADM + fat + partial dural sturing + BFFE	2.7% (3/110)	2.9% (2/69)	2.4% (1/41)

*NA* not available

### Limitations of the CEFB and the present study

First, harvesting the bone flap is not always sufficient or available due to anatomical variations or tumour invasion, especially in cases of oversized defects extending laterally. Second, coverage of the fascia under the wedging edges of the defect is limited. Several measures could be taken to improve these limitations: 1. During the surgical approach, any potentially usable bone structures should be preserved by avoiding excessive grinding of the microdrill. 2. The bone graft could be tailored into narrow strips and then wedged at intervals into the defect (Fig. 3b). 3. The wedging sides of the defect could be carefully enlarged to expose more dural surface for fascia attaching. 4. The dural defect could be minimized by improving the dural incision design and suturing. Finally, in the worst-case scenario, when all measures turn out to be unassured, the PNSF remains a trustworthy last resort.

Our study was retrospective and had only a limited number of patients. The establishment of a randomized control trial is difficult. The current follow-up period of 6 months was also not sufficient to perform long-term evaluation. We have to claim that due to the diversity of clinical conditions, our group set, baseline control, and outcomes comparison might be underpowered owing to the presence of type II error. The statistical analysis in our study should be viewed and interpreted with caution and only for reference. The emphasis of this article is the descriptive data and experience drawn from our surgical practice. A more rigorous study design and accumulation of cases are required for further assessment of the CEFB technique.

## Conclusion

The study suggested that the new CEFB method has the potential to prevent postoperative CSF leak in EES by providing a rigid buttress integrated with multilayer soft-tissue repair. According to our data, in suitable cases of grade II and even grade III intraoperative CSF leaks, the independent use of the CEFB technique had similar reconstruction efficacy to the conventional PNSF and caused fewer nasal complications, shorter bed-stay time and better patient subjective experience. For grade III leaks with multiple high-risk factors or oversize defects, the CEFB could be compatibly combined with the PNSF to ensure reconstruction outcomes.

## Data Availability

Part of the data has been uploaded to the Chinese Clinical Trial Registry (ChiCTR2100044764) (https://www.chictr.org.cn/searchproj.aspx), and the rest of the data are stored in the hospital encrypted database. The nonconfidential part of the data are available upon request.

## Abbreviations

CEFB: Cruciate embedding fascia-bone flap
PNSF: Pedicle vascularized nasoseptal flap
CSF: Cerebral spinal fluid
EES: Endoscopic endonasal surgery
ISBF: In situ bone flap
LD: Lumbar drainage
BMI: Body mass index
EEA: Endoscopic endonasal approach
EEEA: Extended endoscopic endonasal approach
ADM: Acellular dermal matrix
ICA: Internal carotid artery
CT: Computed tomography
ACA: Anterior cerebral artery
OC: Optic chiasm
BF: Bone flap
FL: Fascia lata
DS: Diaphragma sellae
PS: Pituitary stalk
SC: Surgicel
NP: Nasopore
M: Mucosa
SE: Septum

## References

[1] Park HR, Kshettry VR, Farrell CJ, Lee JM, Kim YH, Won TB, Han DH, Do H, Nyguist G, Rosen M, Kim DG, Evans JJ, Paek SH (2017). Clinical outcome after extended endoscopic endonasal resection of craniopharyngiomas: two-institution experience. World Neurosurg.

[2] Bander ED, Singh H, Ogilvie CB, Cusic RC, Pisapia DJ, Tsiouris AJ, Anand VK, Schwartz TH (2018). Endoscopic endonasal versus transcranial approach to tuberculum sellae and planum sphenoidale meningiomas in a similar cohort of patients. J Neurosurg.

[3] Cavallo LM, de Divitiis O, Aydin S, Messina A, Esposito F, Iaconetta G, Talat K, Cappabianca P, Tschabitscher M (2008). Extended endoscopic endonasal transsphenoidal approach to the suprasellar area: anatomic considerations–part 1. Neurosurgery.

[4] Cavallo LM, Messina A, Esposito F, de Divitiis O, Dal Fabbro M, de Divitiis E, Cappabianca P (2007). Skull base reconstruction in the extended endoscopic transsphenoidal approach for suprasellar lesions. J Neurosurg.

[5] Elshazly K, Kshettry VR, Farrell CJ, Nyquist G, Rosen M, Evans JJ (2018). Clinical outcomes after endoscopic endonasal resection of giant pituitary adenomas. World Neurosurg.

[6] Garcia-Navarro V, Anand VK, Schwartz TH (2013). Gasket seal closure for extended endonasal endoscopic skull base surgery: efficacy in a large case series. World Neurosurg.

[7] Gardner PA, Kassam AB, Thomas A, Snyderman CH, Carrau RL, Mintz AH, Prevedello DM (2008). Endoscopic endonasal resection of anterior cranial base meningiomas. Neurosurgery.

[8] Hara T, Akutsu H, Yamamoto T, Tanaka S, Takano S, Ishikawa E, Matsuda M, Matsumura A (2015). Cranial base repair using suturing technique combined with a mucosal flap for cerebrospinal fluid leakage during endoscopic endonasal surgery. World Neurosurg.

[9] Ishikawa T, Takeuchi K, Nagata Y, Choo J, Kawabata T, Ishizaki T, Wakabayashi T (2018). Three types of dural suturing for closure of CSF leak after endoscopic transsphenoidal surgery. J Neurosurg.

[10] Juraschka K, Khan OH, Godoy BL, Monsalves E, Kilian A, Krischek B, Ghare A, Vescan A, Gentili F, Zadeh G (2014). Endoscopic endonasal transsphenoidal approach to large and giant pituitary adenomas: institutional experience and predictors of extent of resection. J Neurosurg.

[11] Koutourousiou M, Fernandez-Miranda JC, Stefko ST, Wang EW, Snyderman CH, Gardner PA (2014). Endoscopic endonasal surgery for suprasellar meningiomas: experience with 75 patients. J Neurosurg.

[12] Marigil Sanchez M, Karekezi C, Almeida JP, Kalyvas A, Castro V, Velasquez C, Gentili F (2019). Management of giant pituitary adenomas: role and outcome of the endoscopic endonasal surgical approach. Neurosurg Clin N Am.

[13] Komotar RJ, Starke RM, Raper DM, Anand VK, Schwartz TH (2012). Endoscopic endonasal compared with microscopic transsphenoidal and open transcranial resection of giant pituitary adenomas. Pituitary.

[14] Hadad G, Bassagasteguy L, Carrau RL, Mataza JC, Kassam A, Snyderman CH, Mintz A (2006). A novel reconstructive technique after endoscopic expanded endonasal approaches: vascular pedicle nasoseptal flap. Laryngoscope.

[15] Lavigne P, Faden DL, Wang EW, Snyderman CH (2018). Complications of nasoseptal flap reconstruction: a systematic review. J Neurol Surg B Skull Base.

[16] Alobid I, Enseñat J, Mariño-Sánchez F, Rioja E, de Notaris M, Mullol J, Bernal-Sprekelsen M (2013). Expanded endonasal approach using vascularized septal flap reconstruction for skull base tumors has a negative impact on sinonasal symptoms and quality of life. Am J Rhinol Allergy.

[17] Tam S, Duggal N, Rotenberg BW (2013). Olfactory outcomes following endoscopic pituitary surgery with or without septal flap reconstruction: a randomized controlled trial. Int Forum Allergy Rhinol.

[18] Jin B, Wang XS, Huo G, Mou JM, Yang G (2020). Reconstruction of skull base bone defects using an in situ bone flap after endoscopic endonasal transplanum-transtuberculum approaches. Eur Arch Otorhinolaryngol.

[19] Conger A, Zhao F, Wang X, Eisenberg A, Griffiths C, Esposito F, Carrau RL, Barkhoudarian G, Kelly DF (2018). Evolution of the graded repair of CSF leaks and skull base defects in endonasal endoscopic tumor surgery: trends in repair failure and meningitis rates in 509 patients. J Neurosurg.

[20] Esposito F, Dusick JR, Fatemi N, Kelly DF (2007). Graded repair of cranial base defects and cerebrospinal fluid leaks in transsphenoidal surgery. Oper Neurosurg (Hagerstown).

[21] Wengier A, Ram Z, Warshavsky A, Margalit N, Fliss DM, Abergel A (2019). Endoscopic skull base reconstruction with the nasoseptal flap: complications and risk factors. Eur Arch Otorhinolaryngol.

[22] Turri-Zanoni M, Zocchi J, Lambertoni A, Giovannardi M, Karligkiotis A, Battaglia P, Locatelli D, Castelnuovo P (2018). Endoscopic endonasal reconstruction of anterior skull base defects: what factors really affect the outcomes?. World Neurosurg.

[23] Sun I, Lim JX, Goh CP, Low SW, Kirollos RW, Tan CS, Lwin S, Yeo TT (2018). Body mass index and the risk of postoperative cerebrospinal fluid leak following transsphenoidal surgery in an Asian population. Singapore Med J.

[24] Lee JY, Barroeta JE, Newman JG, Chiu AG, Venneti S, Grady MS (2013). Endoscopic endonasal resection of anterior skull base meningiomas and mucosa: implications for resection, reconstruction, and recurrence. J Neurol Surg A Cent Eur Neurosurg.

[25] Oakley GM, Christensen JM, Winder M, Jonker BP, Davidson A, Steel T, Teo C, Harvey RJ (2018). Collagen matrix as an inlay in endoscopic skull base reconstruction. J Laryngol Otol.

[26] Prickett KK, Wise SK (2013). Grafting materials in skull base reconstruction. Adv Otorhinolaryngol.

[27] Nishioka H, Izawa H, Ikeda Y, Namatame H, Fukami S, Haraoka J (2009). Dural suturing for repair of cerebrospinal fluid leak in transnasal transsphenoidal surgery. Acta Neurochir (Wien).

[28] Heng L, Zhang S, Qu Y (2020). Cross-reinforcing suturing and intranasal knotting for dural defect reconstruction during endoscopic endonasal skull base surgery. Acta Neurochir (Wien).

[29] Singh H, Essayed WI, Schwartz TH (2020). Endoscopic technology and repair techniques. Handb Clin Neurol.

[30] Zhao D, Tao S, Zhang D, Qin M, Bao Y, Wu A (2018). “Five-layer gasket seal” watertight closure for reconstruction of the skull base in complex bilateral traumatic intraorbital meningoencephaloceles: a case report and literature review. Brain Inj.

[31] Hu F, Gu Y, Zhang X, Xie T, Yu Y, Sun C, Li W (2015). Combined use of a gasket seal closure and a vascularized pedicle nasoseptal flap multilayered reconstruction technique for high-flow cerebrospinal fluid leaks after endonasal endoscopic skull base surgery. World Neurosurg.

[32] Kassam AB, Prevedello DM, Carrau RL, Snyderman CH, Thomas A, Gardner P, Zanation A, Duz B, Stefko ST, Byers K, Horowitz MB (2011). Endoscopic endonasal skull base surgery: analysis of complications in the authors’ initial 800 patients. J Neurosurg.

[33] Thorp BD, Sreenath SB, Ebert CS, Zanation AM (2014). Endoscopic skull base reconstruction: a review and clinical case series of 152 vascularized flaps used for surgical skull base defects in the setting of intraoperative cerebrospinal fluid leak. Neurosurg Focus.

